# Anterior mediastinal tracheostomy – a salvage procedure for tracheal necrosis after thyroidectomy for medullary thyroid cancer: a case-report

**DOI:** 10.1186/s12957-025-03653-0

**Published:** 2025-01-16

**Authors:** Hanan Hemead, Akshay J. Patel, Hannah Jesani, Sajith Kumar, Irfan Ahmed, Sat Parmar, Robert Warner, Neil Sharma, Maninder S. Kalkat

**Affiliations:** 1 Department of Thoracic Surgery, University Hospitals Birmingham, Birmingham, UK; 2 Department of Cardiothoracic Surgery, Alexandria University, Alexandria, Egypt; 3 Institute of Immunology and Immunotherapy, University of Birmingham, Vincent Drive, Edgbaston, Birmingham, B15 2TT UK; 4 Department of Thoracic Anaesthesia, University Hospitals Birmingham, Birmingham, UK; 5 Department of Maxillofacial Surgery, University Hospitals Birmingham, Birmingham, UK; 6 Department of Plastic Surgery, University Hospitals Birmingham, Birmingham, UK; 7 Department of Otolaryngology, Head and Neck Surgery, University Hospitals Birmingham, Birmingham, UK

**Keywords:** Medullary Thyroid Carcinoma (MTC), Anterior Mediastinal Tracheostomy (AMT), Tracheal Necrosis, Pharyngolaryngectomy, Neo-cervical Oesophagus

## Abstract

A 34-year-old male patient with recently diagnosed with medullary thyroid carcinoma underwent total thyroidectomy and radical neck dissection, requiring sharp dissection to separate the tumour from the trachea. He required post operative intubation due to bilateral vocal cord paralysis. He developed ischaemic necrosis of the upper two thirds of the trachea presenting with marked surgical emphysema and an infective wound. The wound was opened, drained and an endotracheal tube was negotiated through the sloughed trachea into the distal intrathoracic trachea with the cuff inflated just above the carina. This complication was managed with total pharyngo-laryngectomy, anterior deep mediastinal tracheostomy and construction of a neo-cervical oesophagus with a free lateral thigh fascio-cutaneous flap. This case highlights the potential complications of a procedure, perseverance, collaboration amongst various disciplines and teamwork for treating a rare and complex condition. The patient was discharged and has had an excellent recovery with good quality of life over two years of follow up.

## Introduction

Anterior mediastinal tracheostomy (AMT) was traditionally performed to secure the airway in cervicomediastinal cancers with a limited length of trachea available for construction of a tracheostoma in the cervical position. The procedure is technically demanding and is associated with high morbidity and mortality. Innominate rupture, mediastinitis and fistulisation are known complications in early case series [1].

Over the years, the refinement of the technique and cumulative experience have improved the procedure outcomes in both the short and long-term. AMT has rapidly become a feasible adjunct for airway management in complex cervicomediastinal malignancies which in our case, was medullary thyroid cancer (MTC). MTC is a rare neuroendocrine disease arising from the parafollicular cells and comprises around 5% of thyroid cancers [2]. It has a strong genetic association and can occur as part of multiple neuroendocrine syndrome 2 (MEN2) or sporadically. Total thyroidectomy with resection of locoregional metastasis offers the best prognosis and potential for complete cure in the absence of distant metastases. Redo neck surgery can still be considered in recurrences or metastatic disease if prognostic benefit can be reasonably achieved [2, 3]. We present a case, where during thyroidectomy for medullary thyroid cancer, inadvertent operative devascularization of the trachea resulted in tracheal injury with less than 5 cm of viable distal trachea. A deep mediastinal tracheostomy was performed, utilizing A pedicled myocutaneous right pectoralis major muscle flap to create a neo-tracheal stoma.

## Case report

**Fig. 1:**
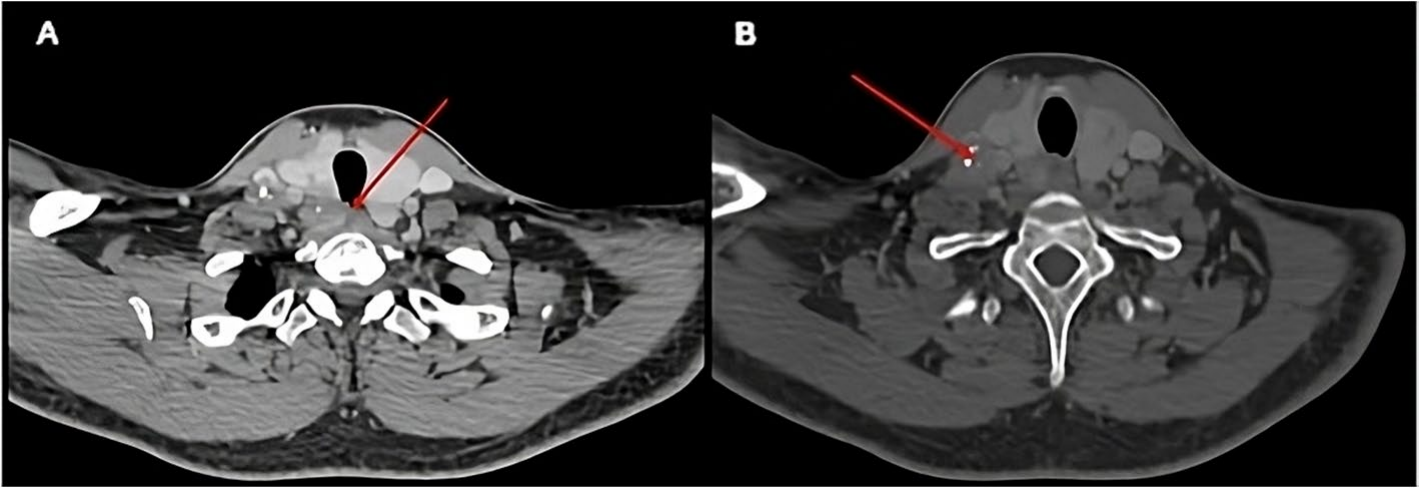
Axial section of CT neck/thorax; red arrow highlights invasion of mediastinal mass into posterior trachea (**A**). Level IV lymph node compressing the right internal jugular vein (**B**)

A thirty-five-year-old male patient underwent elective total thyroidectomy and bilateral level 2–7 neck dissection for a primary right medullary thyroid cancer with preoperative right vocal cord palsy, a large conglomerate nodal mass in right level 4/6 and a solitary metastatic deposit in the L2 lumbar vertebra. Figure 1 demonstrates the pre-operative CT findings. It was determined after multidisciplinary team (MDT) discussion to proceed down a radical route and offer surgery to the neck with stereotactic ablative radiotherapy (SABR) to the spinal lesion.

**Fig. 2:**
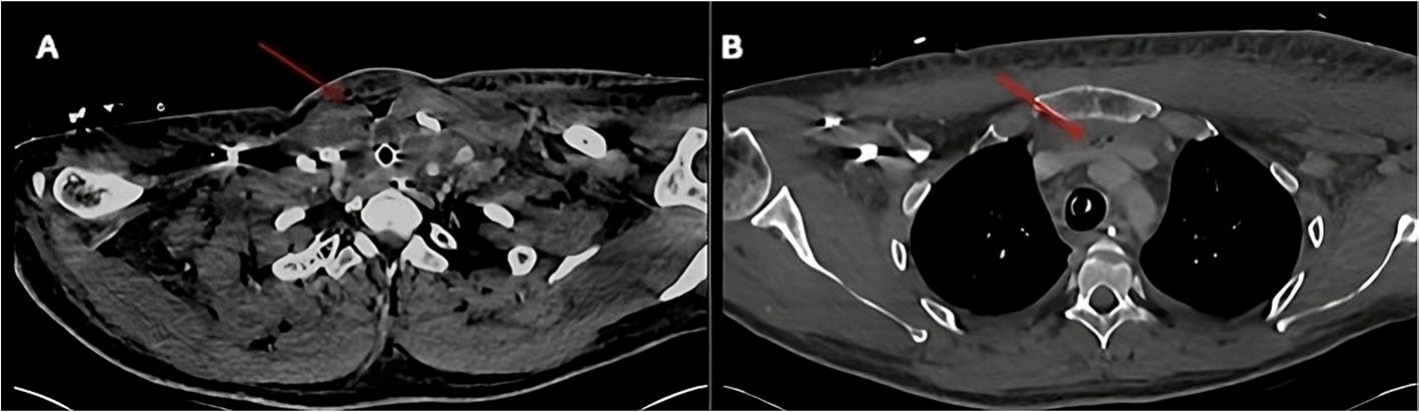
Post-operative CT scan on day 5 (axial section) demonstrates collections in the surgical bed (red arrow) (**A**). Fluid and gas locules within the retrosternal space (red arrow – panel **B**). Collection extending into the superior mediastinum (**B**)

Intraoperatively, the primary tumour was grossly invading the trachea, oesophagus, tracheoesophageal groove, internal jugular vein and thyrocervical trunk with obliteration of planes. Extensive dissection was performed, and a decision was made to “shave” off the tumour from the trachea and treat the residual disease with systemic treatment. During the dissection of left level 6, the signal was lost on the left recurrent laryngeal nerve. Subsequently, the patient failed extubation due to bilateral cord palsy and was re-intubated immediately in operating theatre. On the fifth postoperative day, he developed neck cellulitis, surgical emphysema and sepsis (Fig. 2). Wound exploration revealed an infective collection and a large defect in the cervical trachea with a sloughed wall with the endotracheal tube being exposed in the neck. Intraoperatively, there was bubbling and frank pus within the neck wound and patient was difficult to ventilate. When the endotracheal tube was pushed further, the bubbling stopped and the leak improved. This was suggestive of tracheal perforation on the right side around the level of the 4th ring. The tube was secured distally with the cuff positioned just above the carina and drainage with debridement was performed. Four days later, the wound was re-explored and on inspection a 180-degree tracheal defect was identified at very low level in the neck. Tissues were very friable and unhealthy and attempts of direct repair failed. Decision was made to insert corrugated drains and keep patient intubated until a further plan is decided after MDT discussion.

**Fig. 3:**
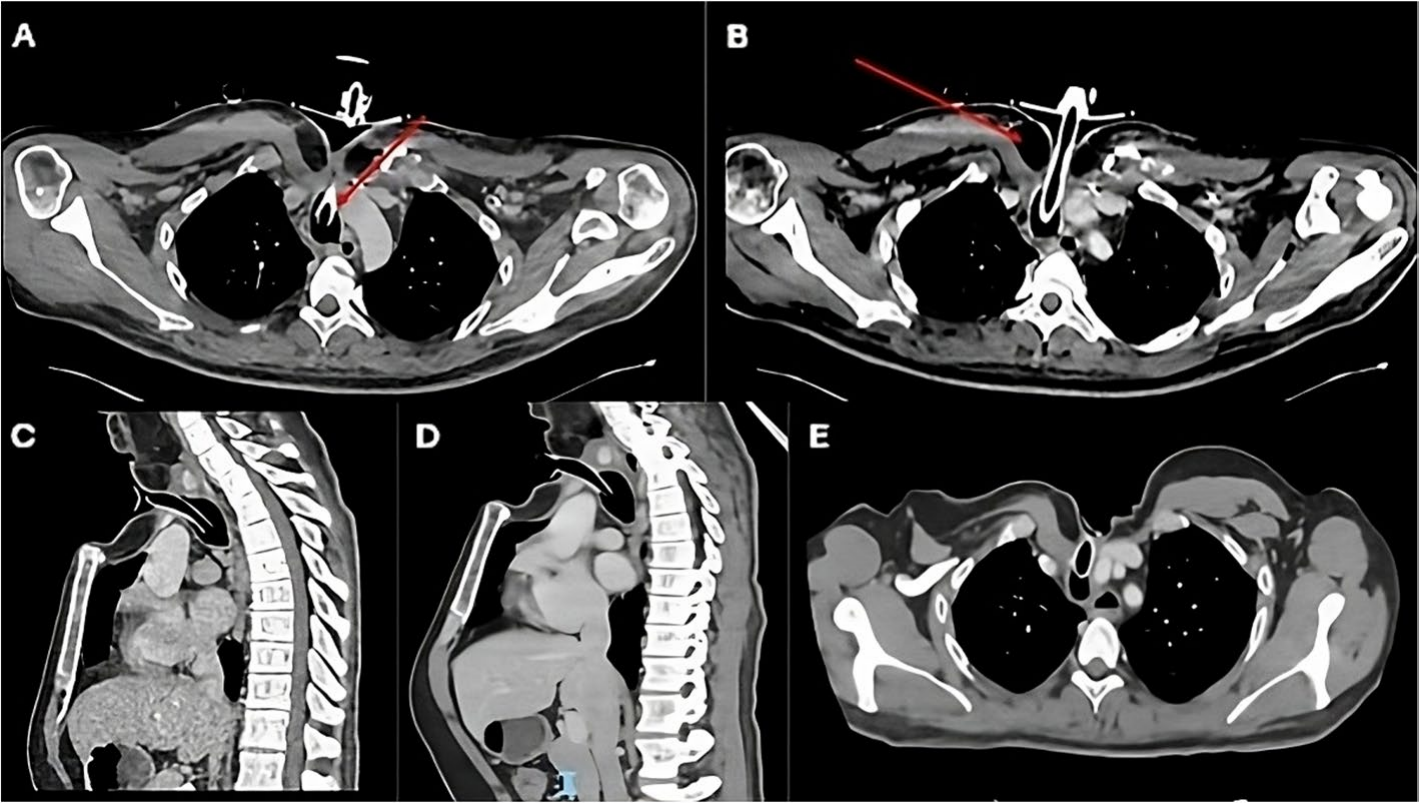
Post-operative CT scan following AMT; **A** and **B** demonstrate the mediastinal tracheostomy, through the circular opening in the Pectoralis Major flap (red arrow). Sagittal sections (**C** and **D**) demonstrating tracheostomy tube entering distal trachea through the muscle flap. Axial section below level of the tracheostoma (panel E)

**Fig. 4:**
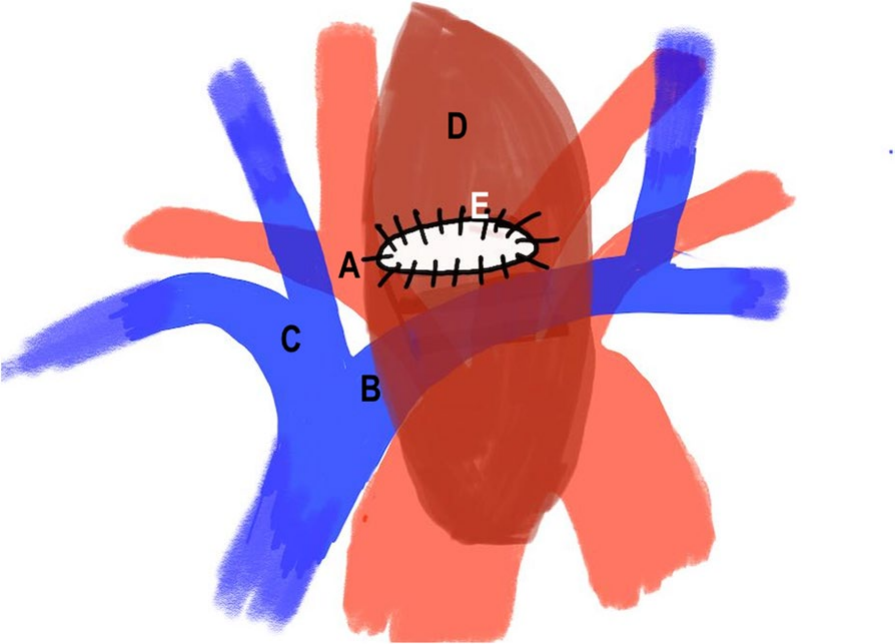
Schematic illustration of the mediastinal tracheostomy stump sutured to pectoralis muscle flap and its relation to innominate vessels. **A** Innominate artery (**B**) Left innominate vein. **C** Right innominate vein (**D**) Pectoralis muscle flap (**E**) Tracheostomy stump

**Fig. 5:**
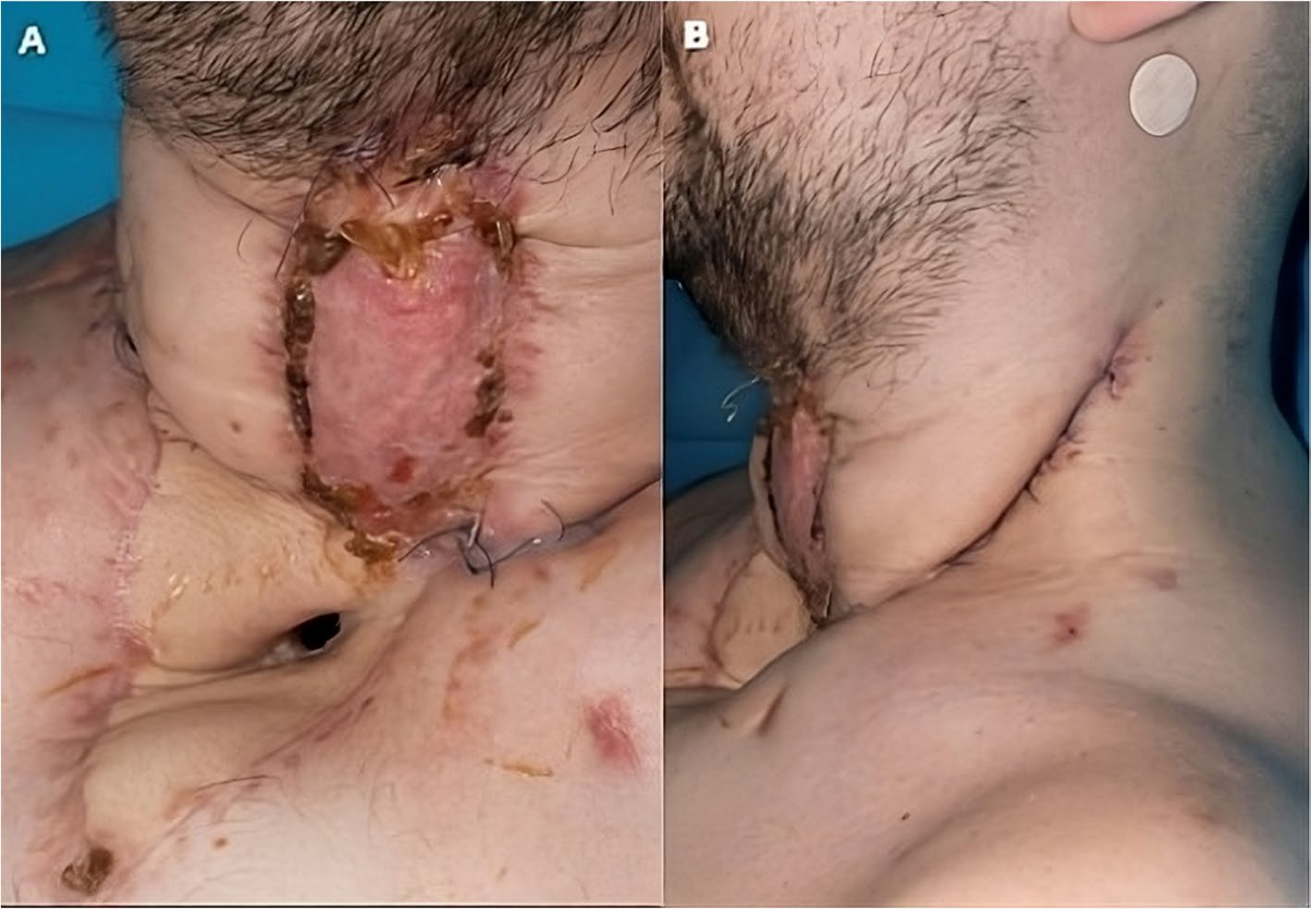
Early post-operative appearances four weeks post discharge from hospital; AP view (**A**), left lateral view (**B**)

In view of the bilateral cord palsy, residual tumour in relation to the cervical oesophagus and the damaged trachea, the MDT discussion advised laryngo-pharyngo-oesophagectomy, securing the airway, followed by oesophageal substitution. Surgery was performed four days later. The pharyngo-laryngectomy along with resection of damaged trachea and cervical oesophagus was performed, thus not only removed the dysfunctional larynx, but achieved tumour clearance. The manubrium with the attached clavicles, first and second ribs bilaterally were all excised to expose the middle mediastinum and viable distal third of the trachea. The distal third of viable trachea was mobilized, ensuring preservation of blood supply and was transposed slightly inferior and lateral to the innominate artery (Fig. 4). The right pedicled pectoralis major muscle flap was raised based on its thoracoacromial blood supply, the entire muscle with a skin island large enough to cover the defect and extend into the mediastinum was harvested for reconstruction of cervical and mediastinal defects. A circular stoma was fashioned in the middle part of the skin island and across the flap. The tracheal remnant was guided through this hole and the tracheal stump was sutured to the muscle and skin using interrupted 4/0 PDS sutures in two layers. The muscle layer of the flap was interposed between the trachea and the innominate artery. The flap was dropped into the mediastinum, ensuring no tension on the tracheal stoma (Fig. 3). Figure 4 shows a schematic drawing of tracheostomy secured to pectoralis muscle flap and its relations to innominate vessels. The cervical oesophagus was reconstructed using a free jejunal graft at the same sitting, but this failed due to poor circulation. A cervical oesophagostomy was performed, and patient was transferred back to intensive care mechanically ventilated. In order to minimise pressure trauma to the tracheal stoma, a modified adjustable length Bivona tracheostomy tube was secured using an Anchorfast strap stuck to the anterior chest wall, holding the tube in a vertical position. Patient movement/turning was only done as strictly necessary. This was maintained until the stoma had healed and the patient woken up, at which point a soft laryngectomy tube was place. Total parenteral nutrition was started on the first postoperative day and changed to enteral nutrition through the jejunostomy tube. The patient was weaned off ventilatory support and transferred to the ward one week later. The laryngo-oesophageal reconstruction was performed electively one month later, using an anterolateral fasciocutaneous free flap (ALT) with vascular reconstruction. Patient was extubated, stepped down to the ward in the second postoperative day and discharged home two weeks later. The patient was discharged home after two weeks. Figure 5 demonstrates the early post-operative appearances in the outpatient clinic four weeks following discharge. At 3 years post operatively, the patient has shown no sign of locoregional recurrence. He has returned to work and phonates well using a Servox electrolarynx. He manages a full diet.

## Discussion

AMT is used sparingly in cases with advanced cervicomediastinal malignancies with tracheal involvement or stomal recurrence after laryngeal resection. Historically, the procedure carries high morbidity and mortality for several reasons: tissue friability, fibrosis due to previous irradiation, stump stenosis/necrosis, extensive dissection, poor vascularisation which leads to early complications such as vascular rupture as well as bony defects which disrupt the chest wall mechanics and lead to respiratory complications [1, 4, 5]. The procedure is technically challenging as it requires multidisciplinary team management and optimal postoperative care. The AMT was associated with a high incidence of fatal haemorrhage from pressure necrosis of the trachea on the innominate artery and stomal dehiscence with compromised airway [6].

The technical modifications have decreased the incidence of these complications. It has been suggested to divide the innominate artery to mitigate against the fatal post operative complications [6]. However, the trachea can be moved inferiorly and to the right of the innominate artery, being cautious not to disturb the blood supply of the trachea. Further protection can be provided by interposing the harvested pectoralis muscle between the trachea and the innominate artery [1, 4, 5]. This additionally provides support of the neo-stoma and reduces the tension on the stoma [4, 5, 7]. The flap also fills in the mediastinal dead space reducing the risk of infection. Over the years, experience showed that to prevent innominate rupture, the vessels should be buttressed with a muscle flap to mitigate against the pressure exerted by the trachea and sutures. In addition, relocating the stump below the innominate artery reduces the apposition with the vessels [8]. The stomal separation can be avoided by meticulous dissection, preserving the vascularity of the residual trachea and ensuring the myocutaneous flap is dropped freely on to the tracheal stoma and sutured without any tension. The removal of the manubrium along with the medial ends of the clavicle and first two ribs facilitate this “drop” and performing a new tracheostoma without tension [9, 10]. Although the pectoralis major flap is commonly used, latissimus dorsi is an alternative if this muscle has been exposed to irradiation [11].

The long-term outcomes of this procedure and survival is dependent on the primary pathology and is under-reported in literature with small case series. Three-year survival is reported to be 44% in cases who underwent AMT for stomal recurrences or thyroid cancers compared to 11% and 0% in cases with primary pathologies of oesophageal and advanced pharyngeal/laryngeal cancers respectively [12].

In the case described, the complications of the primary procedure necessitated application of AMT as a salvage procedure. This procedure was not only necessary for securing the airway of the patient, but also was required due to the dysfunctional larynx and persistence of tumour in the operative field. The salvage procedure was even more challenging due to intense inflammation and pre-existing sepsis, poor circulation which led to failure of the primary jejunal anastomosis. The patient was allowed to recover from the AMT and underwent pharyngo-oesophageal reconstruction using a tubed pedicled anterolateral thigh flap. This conduit for reconstruction has become popular owing to the rich vascularity and ease of harvesting as well as offering an excellent alternative to intestinal flaps which are associated with high morbidity [13]. A similar reconstructive technique was successfully implemented by Liu et al. in recurrent medullary thyroid cancer for restoration of the cervical oesophagus after exenteration with ALT [14].

## Conclusion

This case demonstrates the perils of surgical procedures, with unanticipated complications. The multidisciplinary approach and awareness of surgical possibilities, with proper planning can help salvage the difficult situations. The experience of AMT used as an adjunct in cervicothoracic malignancies was utilized in this case followed by staged reconstruction of the oesophagus with a tubed pedicled anterolateral thigh flap with good outcomes.

## Data Availability

No datasets were generated or analysed during the current study.
